# Analysis of Molar Substitution of Hydroxybutyl Group by Zeisel Reaction in Starch Ethers

**DOI:** 10.3390/molecules26185509

**Published:** 2021-09-11

**Authors:** Xiao-Lei Man, Wei-Kang Peng, Jun Chen, Xue-Li Liu

**Affiliations:** 1 Geosynthetics Applied Research Centre, College of Civil and Architecture Engineering, Chuzhou University, Chuzhou 239012, China; manxl@hhu.edu.cn; 2 College of Material and Chemical Engineering, Chuzhou University, Chuzhou 239012, China; pwkkk68@163.com; 3 College of Biotechnology and Pharmaceutical Engineering, West Anhui University, Lu’an 237012, China; jun.chen06@rainbowfish11000.com; 4 School of Chemistry & Chemical Engineering, Anhui University, Hefei 230601, China

**Keywords:** δ-hydroxybutyl starch, molar substitution, Zeisel gas chromatography, mechanism

## Abstract

A new etherified starch, δ-hydroxybutyl starch (δ-HBS), was prepared by utilising 4-chlorobutan-1-ol as the etherifying reagent. The method of Zeisel gas chromatography for the determination of the molar substitution was described. This technique offers a simple and rapid method for quantitative analysis with reproducible results. Meanwhile, the mechanism of the Zeisel reaction was also investigated.

## 1. Introduction

The increasing industrial importance of hydroxyalkyl starch has created interest in methods for its analysis. Depending on the reaction conditions, the complex substitution pattern differs with respect to DS and MS, and regioselectivity. Among them, the molar degree of substitution (MS) values, i.e., the average number of hydroxyalkyl groups substituted per anhydroglucose unit, directly influence the physicochemical properties [1,2,3,4,5], and convenient methods for its determination have therefore become necessary. 

Several analytical methods have been developed over the years for the determination of the molar substitution ratio of hydroxyalkyl starch. Zeisel gas chromatography has been employed as an analytical technique for the determination of the hydroxyethyl group in hydroxyethyl starch with the quantitative conversion of the substituted alkoxyl unit to the corresponding iodide by reaction with hydriodic acid [6,7,8,9], this technique offers a simple and rapid method for quantitative analysis with reproducible results. Cobler and Samsel [10] and Hodges [11] have analysed the determination of several alkoxyl substitutions in cellulose ethers by gas chromatography; for hydroxypropyl starch, the spectrophotometric (colourimetric) method of Johnson [12,13] is used to determine hydroxypropyl group content and is an official method of the Joint FAO/WHO Expert Committee on Food Additives [1]. Beyond that, Harry-O’Kuru [14] reported a modification of this colourimetric technique for the estimation of the MS of 2-hydroxybutyl starch ether whose preparation was etherified by reaction with epoxybutane [15,16,17,18]. The method involves hydrolysis of the 2-hydroxybutyl group to 1,2-butanediol which in turn is dehydrated to n-butyraldehyde and the enolic form of butenol. The simplicity, low cost, and accuracy of the procedure make it attractive as a routine procedure for industrial application. 

**Figure 1:**
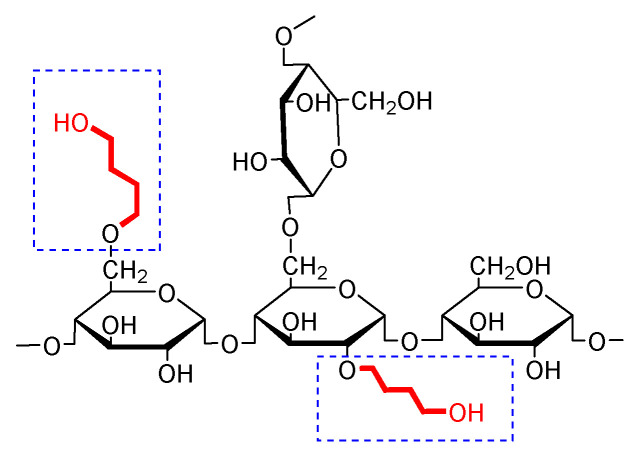
Chemical structure of δ-hydroxybutyl starch.

In recent years, synthesis and characterisation of 2-hydroxybutyl starch have been studied, but there is no similar report on δ-hydroxybutyl starch (δ-HBS), i.e., the hydroxyl group in the terminal of butyl (Scheme S1, Figure 1). Meanwhile, in view of the importance of MS to the physicochemical properties, the present article describes the preparation of a new δ-hydroxybutyl starch and develops a procedure for the analysis of the molar degree of substitution of δ-HBS. In our initial studies, we have established a method for the determination of γ-HPS [19]. Owning to the difference in chemical structure and method applicability [20], we found that the method of spectrophotometry was not suitable to δ-HBS. Thus, we herein report a modification of Zeisel gas chromatography for the estimation of the MS of δ-hydroxybutyl starch ethers.

## 2. Experimental

### 2.1. Materials

Corn starch, δ-hydroxybutyl starch (made in a lab by ourselves), 1-iodobutane, 2-iodobutane, toluene, o-xylene, hydriodic acid (55%), tetrahydrofuran, 1, 4-butanediol (all analytical grade) were obtained from Aladdin Industrial Corporation, Shanghai, China.

### 2.2. Method

The gas chromatography (G5, puri general instrument Co., Ltd., Beijing, China) was equipped with an FID detector, and the capillary chromatographic column was SPB-5 (30 m × 0.32 mm × 0.25 μm). The GC operating condition was as follows: The injector was held at 200 °C, and the initial oven temperature of 60 °C was held for 4 min, which was then increased to 230 °C at 30 °C/min. This temperature was also held for 4 min. The injection volume was 2 µL. The detector temperature was 250 °C.

Iodobutane, toluene, o-xylene were used for GC blank analysis, respectively. Toluene was used as the internal standard. Toluene of 1.0 mL was dissolved in o-xylene and made into a 200 mL solution in a volumetric flask. A set of standards was prepared by injecting 1, 2, 3, 6, 10, 18, 28, and 40 μL of iodobutane into test tubes stoppered with Suba Seals containing 2.0 mL of internal standard solution. The tubes were mixed and shaken vigorously and then allowed to stand for 10 min. The exact weight of iodobutane was recorded. Each solution was used for GC analysis.

Approximately 50 mg of δ-hydroxybutyl starch or unmodified starch, accurately weighed, was transferred to a 25 mL reactive vial containing 200–250 mg of adipic acid. Two millilitres of internal standard solution and 5 mL of hydriodic acid were added to the vial, which was immediately tightly capped with a Mininert valve and weighed. Vials were placed in a preheated block at 140 °C for 10 h. After being heated, the vials were removed from the block, shaken, cooled to room temperature, and reweighed to determine if any loss due to leakage had occurred. Then, 0.1 mL of the upper organic layer was transferred to a sampler vial containing 1.0 mL of o-xylene. The sampler vial was capped tightly and mixed for 5 s. The obtained sample would be used for GC analysis.

### 2.3. Calibration

Calculation of the MS of hydroxybutyl starch is historically based on the butyl oxide unit, C4H8O. The MS ratio, which is defined as the mole fraction of epoxybutane per anhydroglucose unit, is calculated by the following equations:

(1) $$W_{p}=\frac{72.11\times P\times 100}{184.02\times m}\;;\;\mathrm{MS}=\frac{W_{p}}{100-W_{p}}\times \frac{162.14}{72.11}\;$$

The *P* in formula 1 equals the weight of iodobutane and m equals the weight of sample; the values 184.02 and 72.11 in equations equal the molecular weight of iodobutane and C4H8O, and 162.14 equals the molecular weight of anhydroglucose.

**Figure 2:**
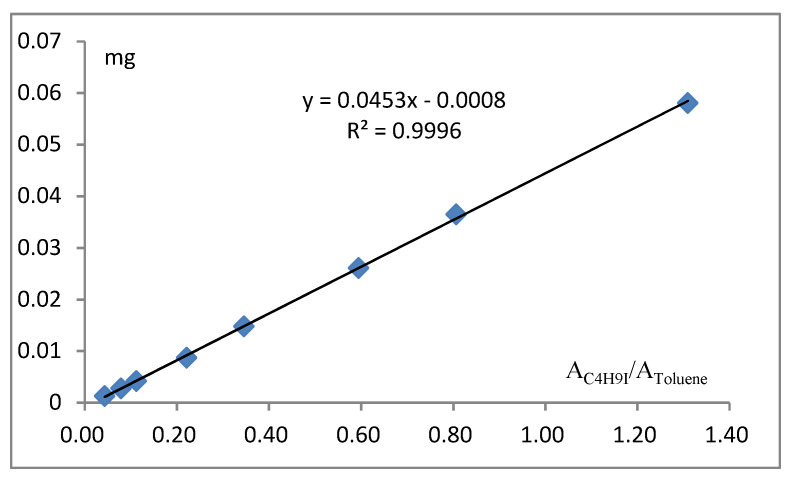
The linear curve of reference substance of 2-iodobutane.

Based on the percent alkoxyl substitution anticipated in the prepared samples, the quantity of respective 2-iodobutane needed for calibration was determined according to the graph in Figure 2. The solutions were analysed by GC for obtained the relative calibration factor between 2-iodobutane and toluene. The calibration curve for butyl iodide in o-xylene ranging in concentration from 1.3 to 58.1 mg/mL was constructed. Relationship of $m_{C4H9I}/m_{Toluene}$ to $A_{C4H9I}/A_{Toluene}$ is shown in Figure 2. $m_{C4H9I}/m_{Toluene}$ is in direct proportion to $A_{C4H9I}/A_{Toluene}$, and the curve almost goes through the zero point. Statistical analysis of the calibration data was performed. All the data points fell on the same calibration line, which had a correlation coefficient of 0.9998. By linear regression, an equation for the calibration curve obtained is as follows:

(2) y=0.0453x−0.0008, R=0.9998 

The repeatability, linear range, and detection limits were studied for the GC method. The RSD for six replicate runs was 1.11% (*n* = 6), which indicates that the method is sufficiently precise. 

## 3. Results and Discussion

### 3.1. Process and Mechanism

**Figure 3:**
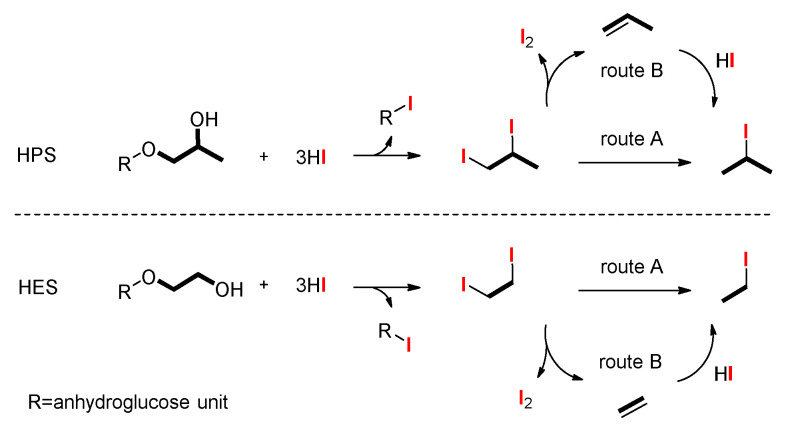
Proposed mechanism for the hydriodic acid decomposition procedure of HPS and HES.

**Figure 4:**
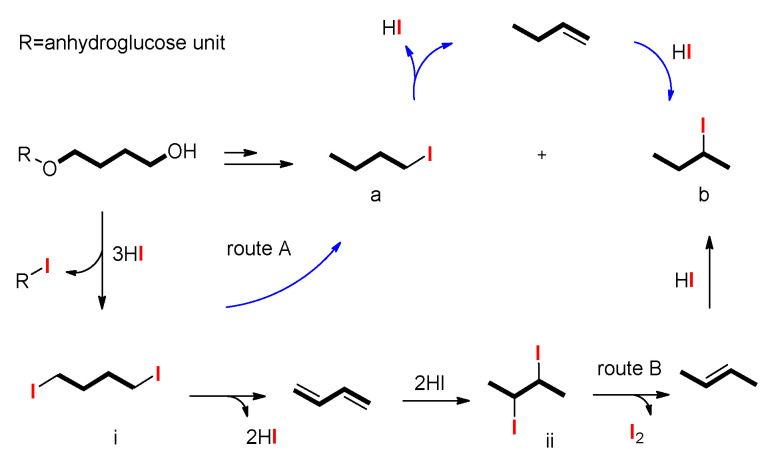
Proposed mechanism of iodobutane generation from δ-HBS. (R=anhydroglucose unit).

**Figure 5:**
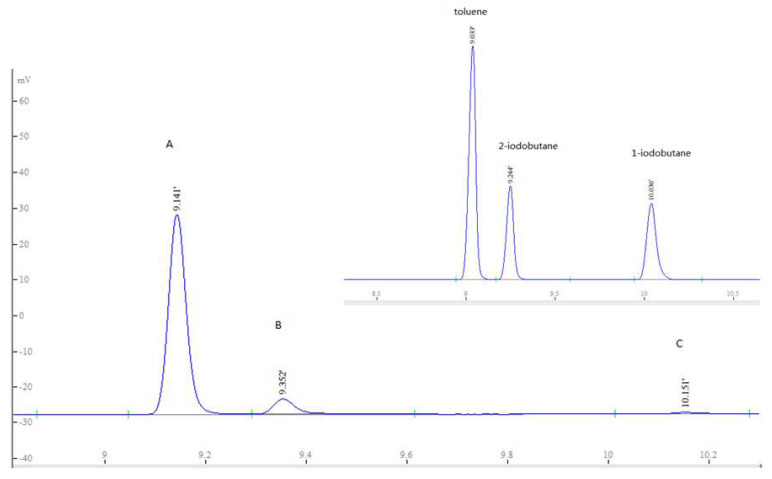
Chromatogram of extracts of the upper (o-xylene) layer of the reaction solution: (**A**) toluene; (**B**) 2-iodobutane; (**C**) 1-iodobutane.

The classical Zeisel distillation method utilises adipic acid to catalyse the hydriodic acid cleavage of the substituted alkoxyl groups quantitatively to their corresponding alkyliodides. Different from the cleavage of hydroxypropyl and hydroxyethyl starch [7,9,21] (Figure 3) or cellulose ethers [11], the procedure of displacement reaction of 4-hydroxybutyl starch ether was slightly complicated (Figure 4). The final reaction products have two parts—namely, 1-iodobutane and 2-iodobutane (Figure 5 and Figures S3–S6). The latter is the major reaction product. As it is mentioned in the mechanism, the first generated diiodo intermediate (i) proceeds through two routes to the desired alkyl iodide. One is acid catalysed and the direct conversion to 1-iodobutane (a) which can be converted to 2-iodobutane (b) by a succession of elimination and addition reaction. Another is the quantitative conversion of alkoxyl unit to butadiene which would be converted to the final 2-iodobutane (b). This conversion proceeds through a postulated vicinal 2,3-diiodo intermediate (ii). Each route can lead to the generation of 2-iodobutane (b).

To gain more insights into the reaction mechanism, a few control reactions were conducted. As shown in Table 1, support for this mechanism was obtained by heating tetrahydrofuran, 1,4-butanediol, in the presence of hydroiodic acid with o-xylene being present (entry 1–2). The result indicated that 2-iodobutane is the main hydrolysis product. Even then, there is still a little vestigial 1-iodobutane in the reaction mixture, and the peak area ratio of 2-iodobutane/1-iodobutane is 48.9:1 and 49.1:1. The exact quantity of the final product can be calculated by gathering 1- and 2-iodobutane. The relative error of the observed means was approximately 4.3% and 4.5%. Meanwhile, 1-iodobutane was also investigated (entry 3–5). Under the same catalytic system, 1- and 2-iodopropane were detected by chromatographing, and it was observed that after 24 h, the conversion of reaction can reach up to 50%. In addition, the gas chromatographic analysis was very rapid, with complete resolution of all peaks within a total retention time of less than 11 min. 

### 3.2. Method Application

The results of analysing three samples of hydroxybutyl starches containing significantly different amounts of hydroxybutyl substitution by the Zeisel-GC technique are shown in Table 2 and Table S1. The molar substitution increased as the volume of etherifying agent added to the reaction mixture increased, while other parameters were kept constant. This observation is consistent with increases in the MS with corresponding increases in the volume of the etherifying agent in the hydroxypropylation of starches [22,23,24].

### 3.3. Characterisation

#### 3.3.1. FTIR Spectroscopy

**Figure 6:**
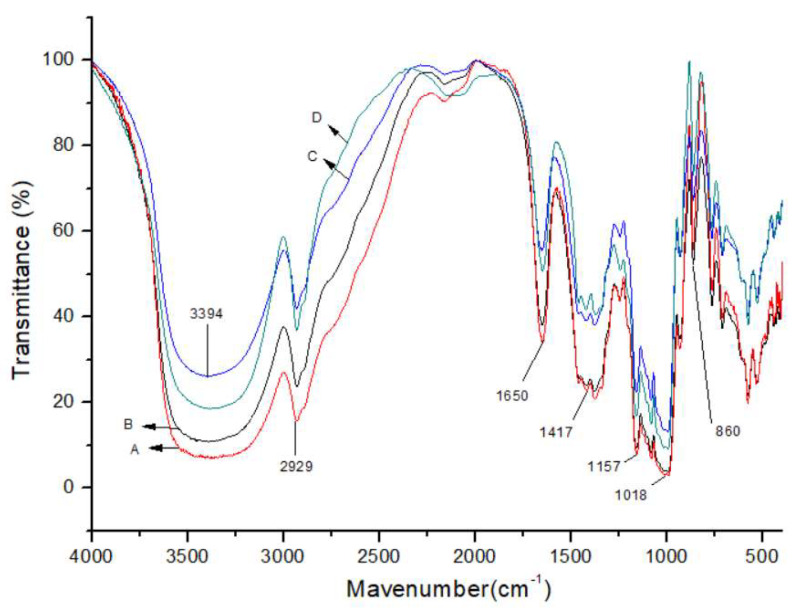
FTIR spectra of δ-HBS at (**D**) MS = 0.12, (**C**) MS = 0.076, (**B**) MS = 0.042, and (**A**) unmodified starch.

A typical FTIR spectrum of δ-HBS is shown in Figure 6, Figures S1 and S2, together with a spectrum of unmodified corn starch. In comparison with unmodified corn starch, the difference between δ-HBS and the starch spectrum was conspicuous. The IR spectrum of δ-HBS showed the distinct absorption bands that appeared at 3394 cm^−1^, which was assigned to stretching vibration of -OH. The absorption bands became narrower and shifted to higher wavenumbers. The weak peak toward 2929 cm^−1^ was attributed to the C-H asymmetric stretching vibration. The high density of -CH_2_ groups was in the δ-HBS polymer structure. A large quantity of -CH_2_ groups was fetched into HBS by etherification, and the peak of the δ-HBS with higher MS at 1417 cm^−1^ which was corresponding to bending vibration of -CH_2_ showed significant change. The absorption peak of the C-O stretching of the ether group was observed at 1157 cm^−1^, as shown in Figure 6. In addition, the absorption peak of C-O-C glycosidic bonds of AGU of starch molecules at 860 cm^−1^ was observed to remain intact, suggesting that the aforementioned molecules were not degraded after etherification. Therefore, the results of FTIR spectra further proved that δ-HBS was synthesised.

#### 3.3.2. NMR Spectroscopy

**Figure 7:**
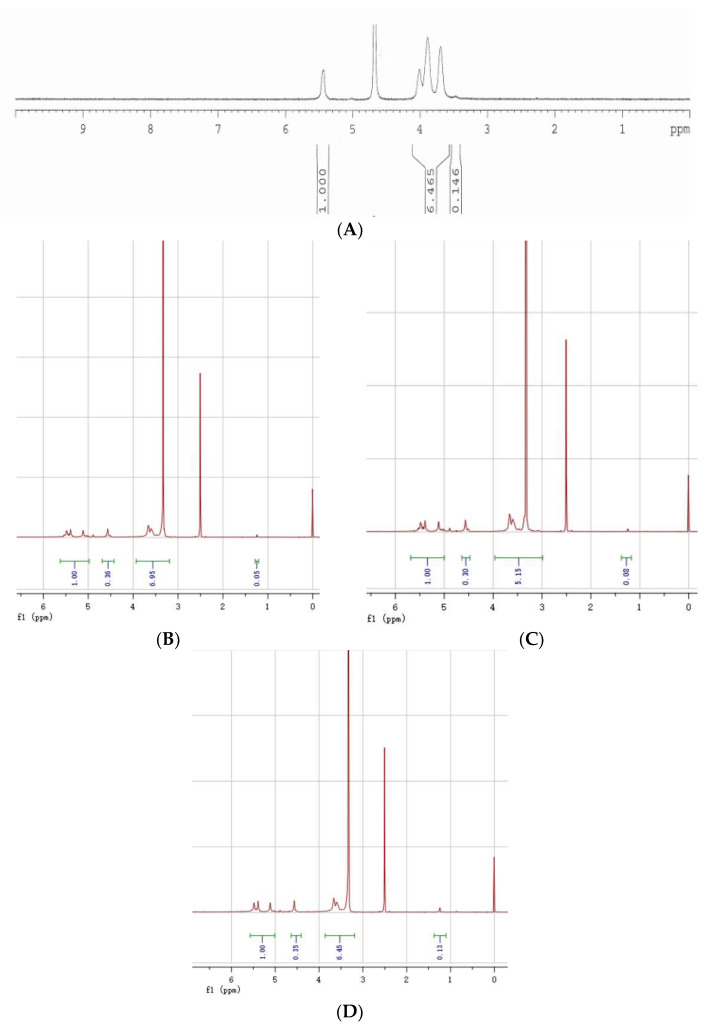
H-NMR spectra of δ-HBS at (**D**) MS = 0.12, (**C**) MS = 0.076, (**B**) MS = 0.042, and (**A**) unmodified starch.

A typical ^1^H NMR spectrum of δ-HBS is shown in Figure 7, together with a spectrum of unmodified corn starch. The ^1^H NMR spectral signals between 3.4 and 4.1 ppm (Figure 7A) corresponded to the six protons of the constituent repeating α-D-glucopyranosyl units (AGUs) of unmodified starch, except for the proton of acetal (O-C(H)-O) observed as a singlet at 5.4 ppm. Figure 7B–D show the peaks between 3.2 and 3.8 were assigned to the six protons of the AGUs and the four protons of O-CH_2_- group from the O-CH_2_-CH_2_-CH_2_-CH_2_-OH group of the substituent. Owing to the introduction of OCH_2_CH_2_CH_2_CH_2_OH, the characteristic peak of the proton of acetal (O-C(H)-O) was shifted and split, resulting in a multiple-peak separation between 5.1 and 5.5 ppm. The clear broadening of anomeric proton’s peak for δ-HBS is due to the substitution at the O-2 position [25,26]. The presence of a spectral signal at 1.24 ppm (Figure 7B–D) confirmed the presence of four methylene protons of the C-CH_2_-CH_2_-C group in -OCH_2_CH_2_CH_2_CH_2_OH. The spectrograms indicated successful etherification.

## 4. Conclusions

A new class of hydroxybutyl starch was synthesised by utilising 4-chlorobutan-1-ol as the etherifying reagent. Zeisel gas chromatography was adopted for the analysis of the MS of δ-HBS. For consideration of the potentials of a relatively new, cheap, underutilised but abundant starch resource, further investigations are currently underway in our laboratory.

## Figures and Tables

**Table 1:** Control experiments.

Entry	Sample ^[a]^	Amt Added ^[b]^/mg	a ^[c]^/mg	b ^[d]^/mg	Theoretical Weight ^[e]^/mg
1	tetrahydrofuran	32.6	1.59	78.00	83.19
2	1,4-butanediol	25.1	0.98	47.93	51.25
3	1-iodobutane	40.1	27.63	10.83	40.1
4 ^[f]^	1-iodobutane	27.8	24.70	1.58	27.8
5 ^[g]^	1-iodobutane	37.5	18.44	17.60	37.5

^[a]^ Unless otherwise stated, the hydriodic acid cleavage reaction condition: 1 mL internal standard solution, 2 mL hydriodic acid (55–58%), 200 mg adipic acid, 140 °C for 12 h; ^[b]^ the mass of sample; ^[c]^ the experimental values of 1-iodobutane(a); ^[d]^ the experimental values of 2-iodobutane (b); ^[e]^ the theoretical weight of iodobutane; ^[f]^ for 6 h; ^[g]^ for 24 h.

**Table 2:** Determination of butoxy groups in hydroxybutyl starch ether.

Sample ^[a]^	Amt Added ^[b]^/mg	Peak Area Ratio ^[c]^	Observed/mg ^[d]^	MS ^[e]^
1	58	0.078	2.74	0.042
2	51	0.112	4.26	0.076
3	56	0.177	7.24	0.120

^[a]^ In-house synthesised δ-hydroxybutyl starch ether; unless otherwise stated, the reaction was carried out in 50 mL solvent using NaOH (0.6 g) and chlorobutanol (1.5 mL, 2.5 mL, 3.5 mL) with corn starch (10 g) at 50 °C for 20 h; ^[b]^ the mass of δ-hydroxybutyl starch ether; ^[c]^ the peak area ratios of $A_{C4H9I}/A_{Toluene}$; ^[d]^ the measured value of $m_{C4H9I}$ by using the equation of calibration curve (2); ^[e]^ using the equation of calibration curve (1).

## Data Availability

Not applicable.

## Supplementary Materials

The following are available online. Supplementary data contains the following information: Synthesis of δ-HBS (Scheme S1), characterizations of δ-HBS using FTIR and ^1^ H NMR (Figures S1 and S2), precision determination (Table S1), gas chromatograms (Figures S3–S6).

## Sample Availability

Samples of the compounds are available from the authors.
