# Supplementary material for: Analysis of Molar Substitution of Hydroxybutyl Group by Zeisel Reaction in Starch Ethers
Source: Molecules. 2021 Sep 11;26(18):5509. doi: 10.3390/molecules26185509 (PMC8465643; doi:10.3390/molecules26185509)
Supplement: Supplementary file 1 [file molecules-26-05509-s001.zip › molecules-1368383-supplementary.pdf]

SUPPORTING INFORMATION

Analysis of Molar Substitution of Hydroxybutyl Group by Zeisel Reaction in starch ethers

Xiao-Lei Man[1], Wei-Kang Peng [2], Jun Chen[3], Xue-Li Liu[24]*

[1] *Geosynthetics Applied Research Centre, College of Civil and Architecture Engineering, Chuzhou University, Anhui 239012, China.*

[2] *College of Material and Chemical Engineering, Chuzhou University, Anhui 239012, China.*

[3]*College of Biotechnology and Pharmaceutical Engineering, West Anhui University, Anhui 237012, China.*

[4]*School of Chemistry and Chemical Engineering, AnHui University, He Fei, 230601, China*

[*] *Correspondence, e-mail: n_xueli@163.com.*

Table of Contents Page

1. General experimental procedure……………………………………………1

2. Experimental data and spectrogram……………………………..…...….. 1

1. Experimental Procedures

1. 1 Synthesis of δ-Hydroxybutyl starch (δ-HBS)

Briefly, corn starch (10g) was suspended in isopropyl alcohol, then the solution was initiated by addition of NaOH(0.6g) for 1h under sterring. A predetermined amount of 4-chlorobutan-1-ol (1.5mL, 2.5mL, 3.5mL) added to the flask. The reaction was carried out at 50°C for 20h. Then the suspension was cooled and neutralized to pH 7.0 with 1 M hydrochloric acid. Collected the product by suction filtration and washed on the filter with 95% ethanol aqueous solution three times, and then dried in a vacuum oven.

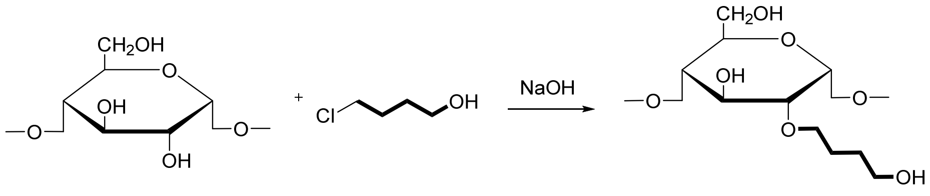

Scheme S1. Synthesis pathway of the δ-Hydroxybutyl starch.

2. Experimental data and spectrogram

2.1 FTIR Spectroscopy of δ-Hydroxybutyl starch

Briefly, A typical FTIR spectrum of δ-HBS is shown in Figure 1, together with a spectrum of unmodified corn starch. In comparison with unmodified corn starch, the difference between δ-HBS and starch spectrum was conspicuous. The IR spectrum of δ-HBS showed the distinct absorption bands appeared at 3394 cm^{-1}, which was assigned to stretching vibration of -OH. The absorption bands became narrower and shifted to higher wave number. The weak peak toward 2929 cm^{-1} was attributed to the C-H asymmetric stretching vibration. High density of $-CH_2$ groups

were in the δ-HBS polymer structure. A large quantity of -CH_2 groups were fetched into HBS by etherification, the peak of the δ-HBS with higher MS at 1417 cm^{-1} which was corresponding to bending vibration of -CH_2 showed significant change. The absorption peak of the C-O stretching of the ether group was observed at 1157 cm^{-1} as shown in Figure 1. In addition, the absorption peak of C-O-C glycosidic bonds of AGU of starch molecules at 860 cm^{-1} was observed to remain intact, suggesting that the aforementioned molecules were not degraded after etherification. Therefore, the results of FTIR spectra further proved that δ-HBS was synthesized.

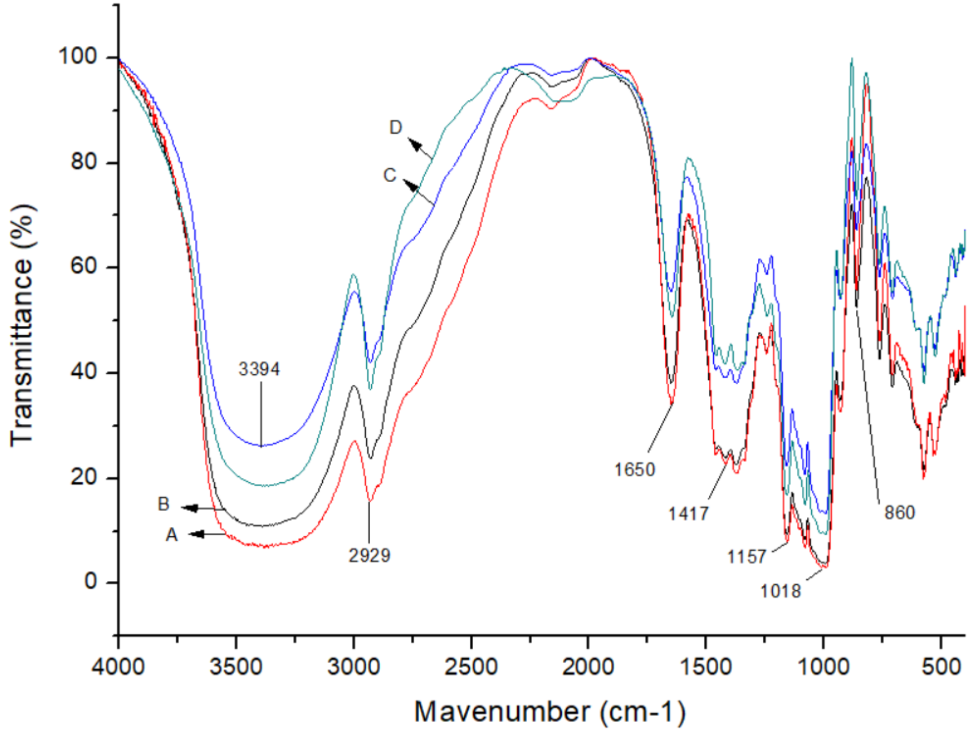

Figure S1. FTIR spectra of δ-HBS at D) MS = 0.12, C) MS = 0.076, B) MS = 0.042, and A) unmodified starch.

2.2 ^{1}H NMR of δ-Hydroxybutyl starch

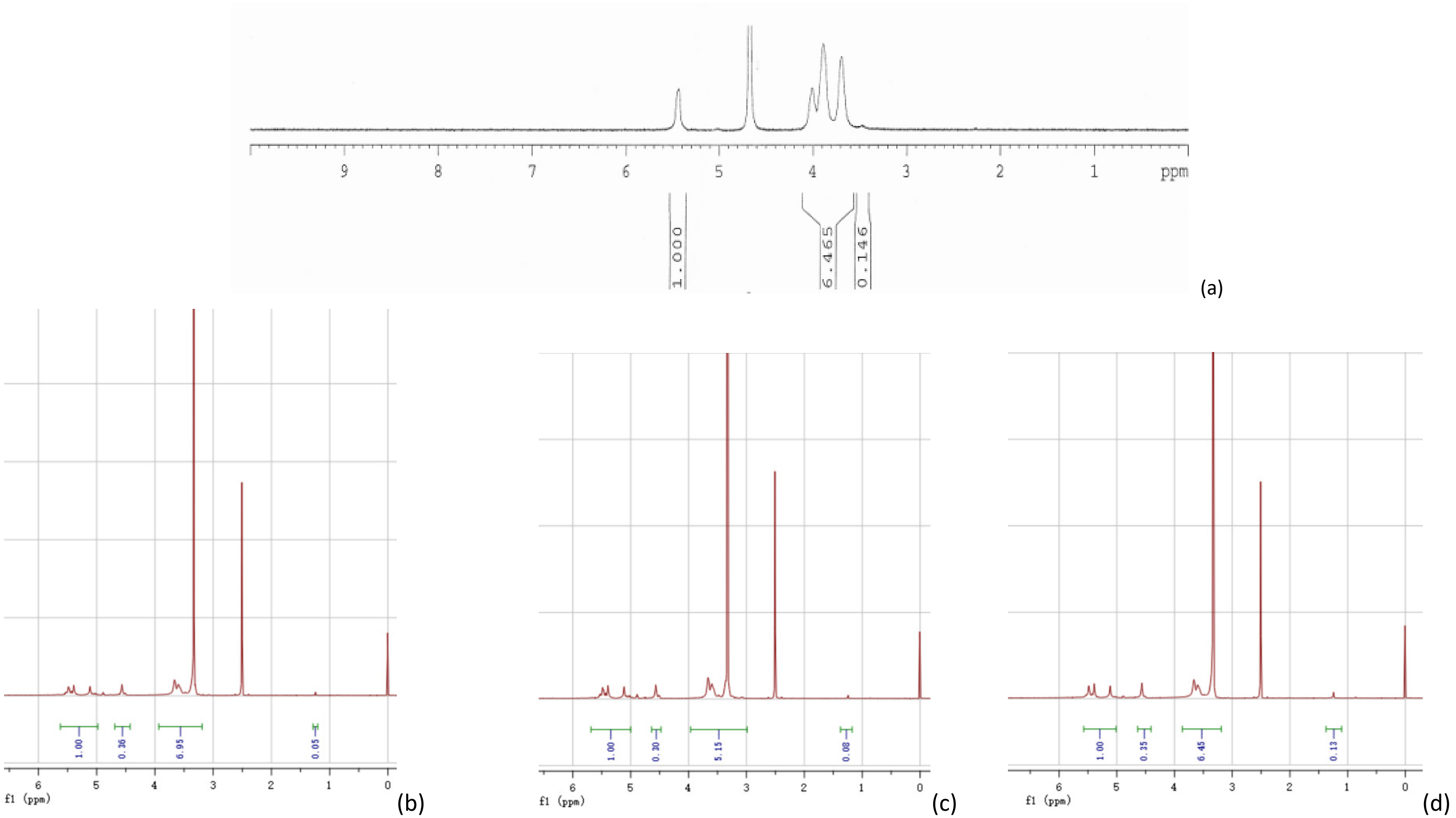

the H-NMR spectrum of unmodified starch

Figure S2. H-NMR spectra of δ-HBS at D) MS = 0.12, C) MS = 0.076, B) MS = 0.042, and A) unmodified starch.

The ^{1}H NMR spectral signals between 3.4 and 4.1 ppm (Fig.2a) corresponded to the protons of the constituent repeating α-D-glucopyranosyl units of unmodified starch, except for the proton of acetal (O-C(H)-O) observed as a singlet at 5.4 ppm. Owing to the introduction of -$OCH_2CH_2CH_2CH_2OH$, the characteristic peak of the proton of acetal (O-C(H)-O) was shifted and split, and the result in multiple-peak separation between 5.1 and 5.5 ppm. The presence of spectral signal at 1.24 ppm (Fig.2b-2d) confirmed the presence of methylene proton in -$OCH_2CH_2CH_2CH_2OH$.

2.3 Gas chromatography

The discussion please see the article.

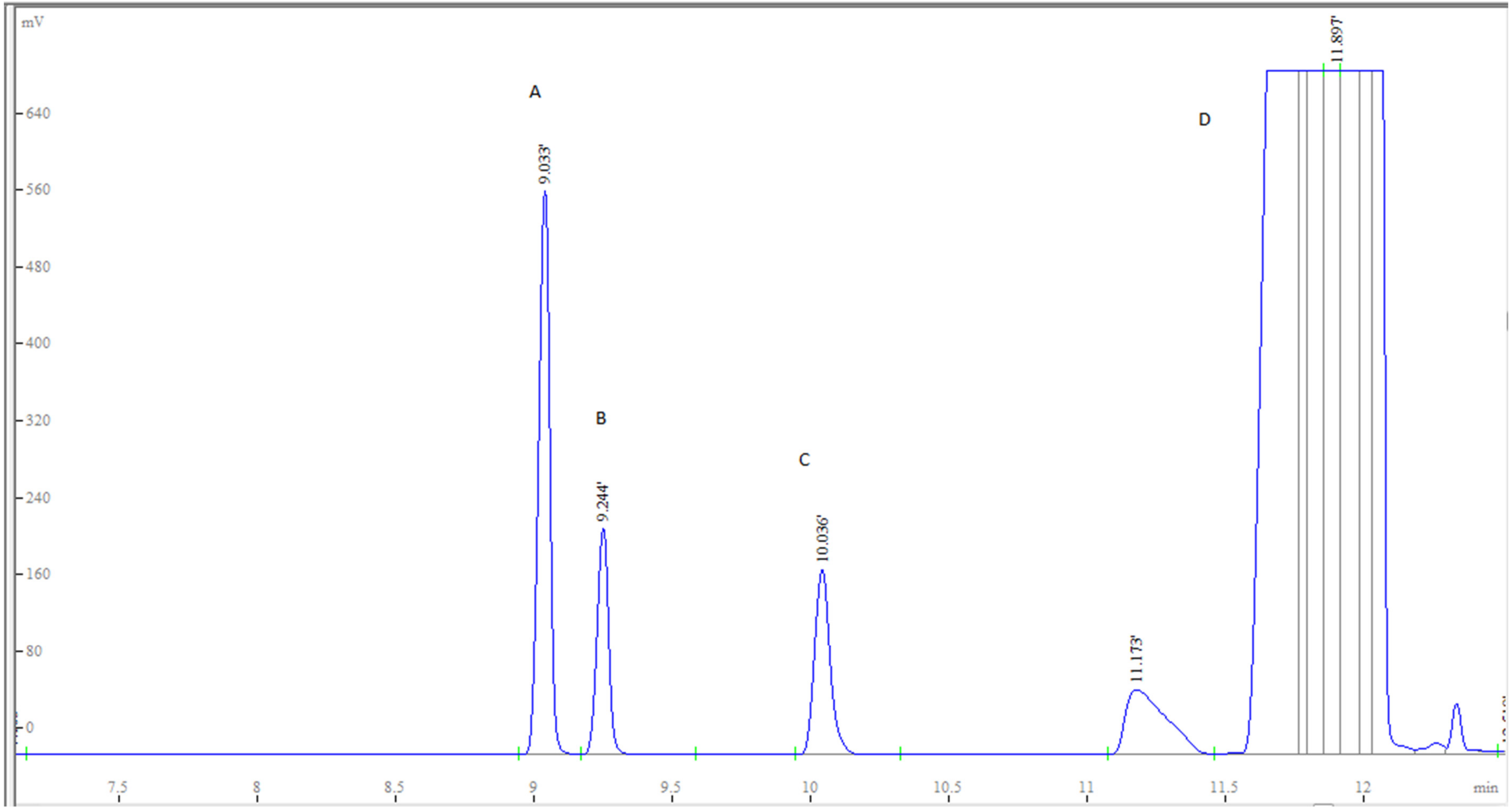

Figure S3. Chromatogram of iodobutanes, toluene and o-xylene solvent. (A) toluene; (B)2-iodobutane; (C)1-iodobutane; (D) o-xylene

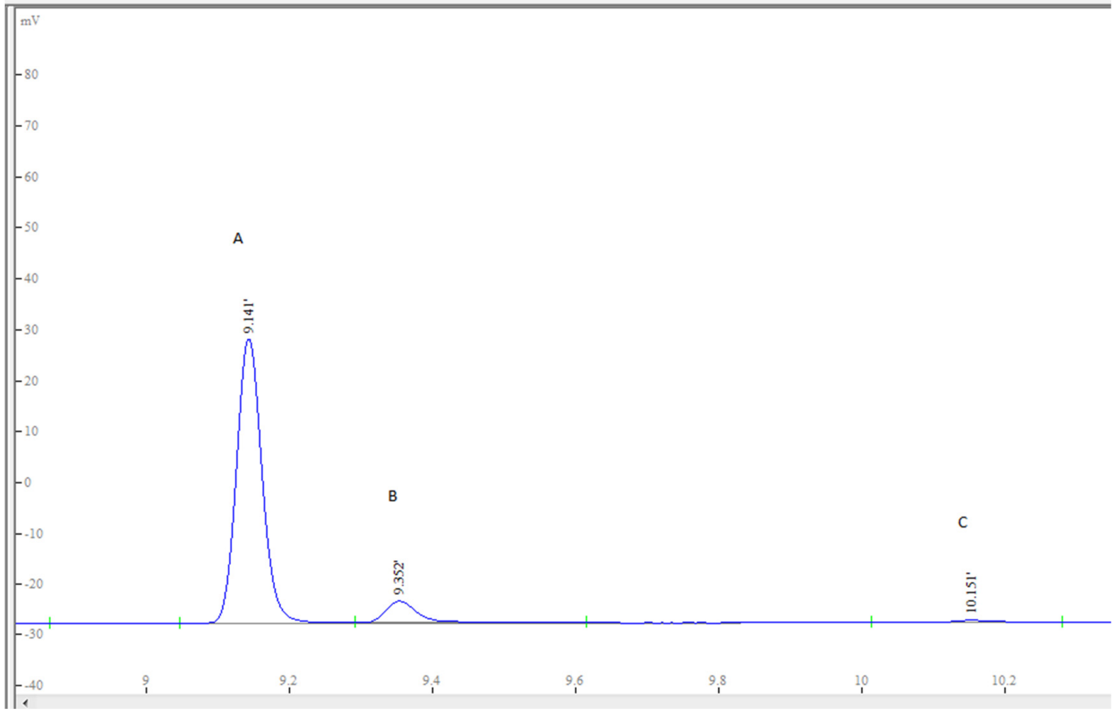

Figure S4. The validation experiment of the final hydrolysis product. (A) toluene; (B)2-iodobutane; (C)1-iodobutane.

By chromatographing the reaction products over the course of the experiment, one can at least partially understand the chemistry of the cleavage. The peak area ratio of B/C is 33.9:1, which indicated that 2-iodobutane is the main hydrolysis product. The exact quantity of final hydrolysis product can be calculated by gathering B and C.

Table S1. Results of the precision determination of the proposed method

No.	1	2	3	4	5	6
$A_{C4H9I}/A_{Toluene}$	0.345	0.344	0.346	0.351	0.341	0352
**average ratio**			0.3465			
**standard deviation**			0.00386221			
**RSD**			1.11			

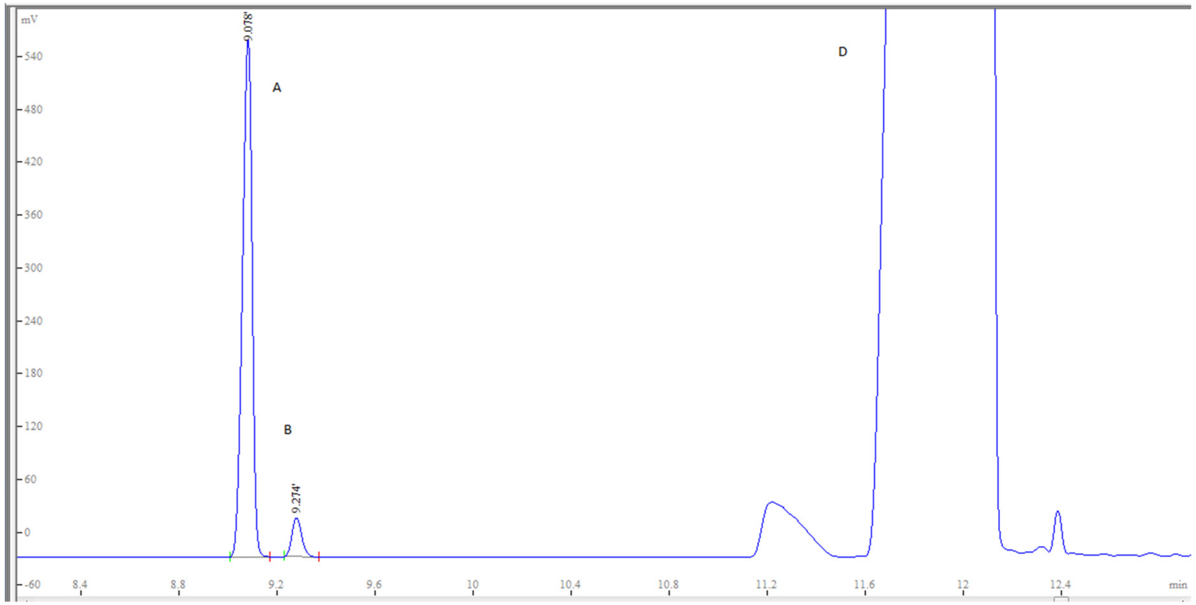

(a)

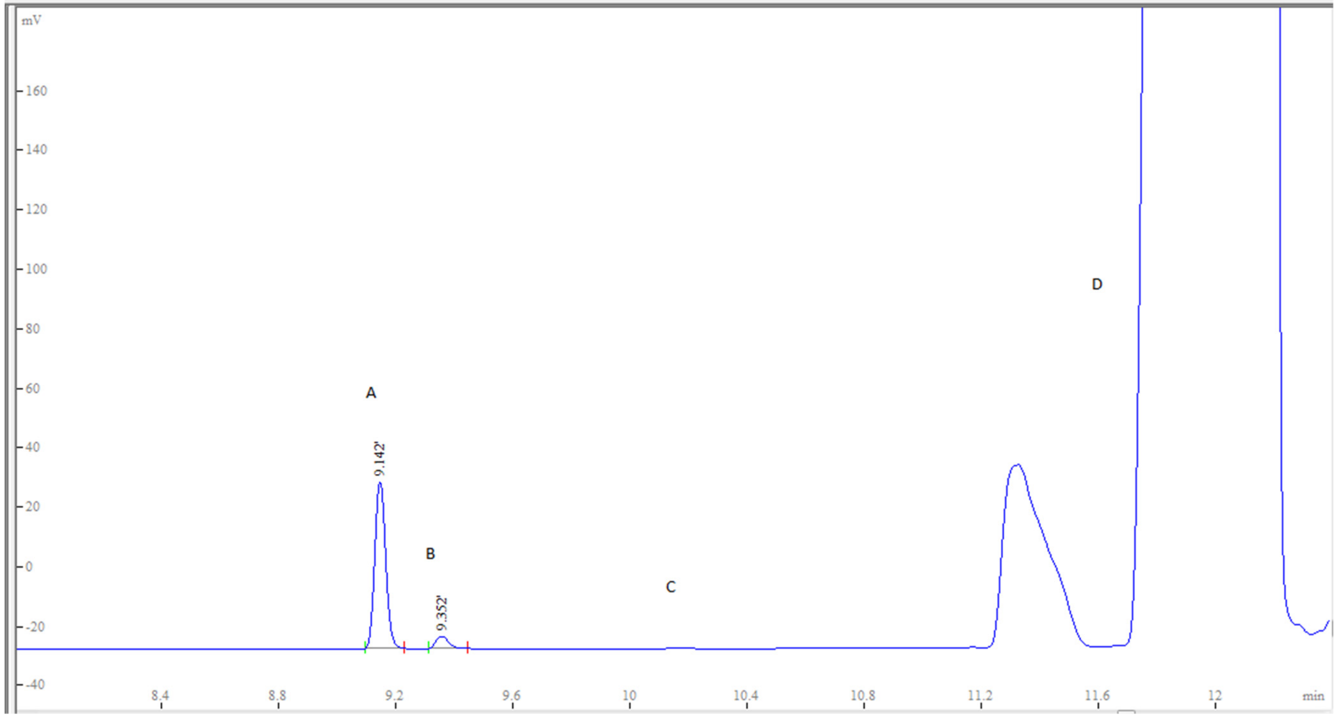
mV
160
140
120
100
80
60
40
20
0
-20
-40
A
9.142'
B
9.352'
C
D
8.4
8.8
9.2
9.6
10
10.4
10.8
11.2
11.6
12
min

(b)

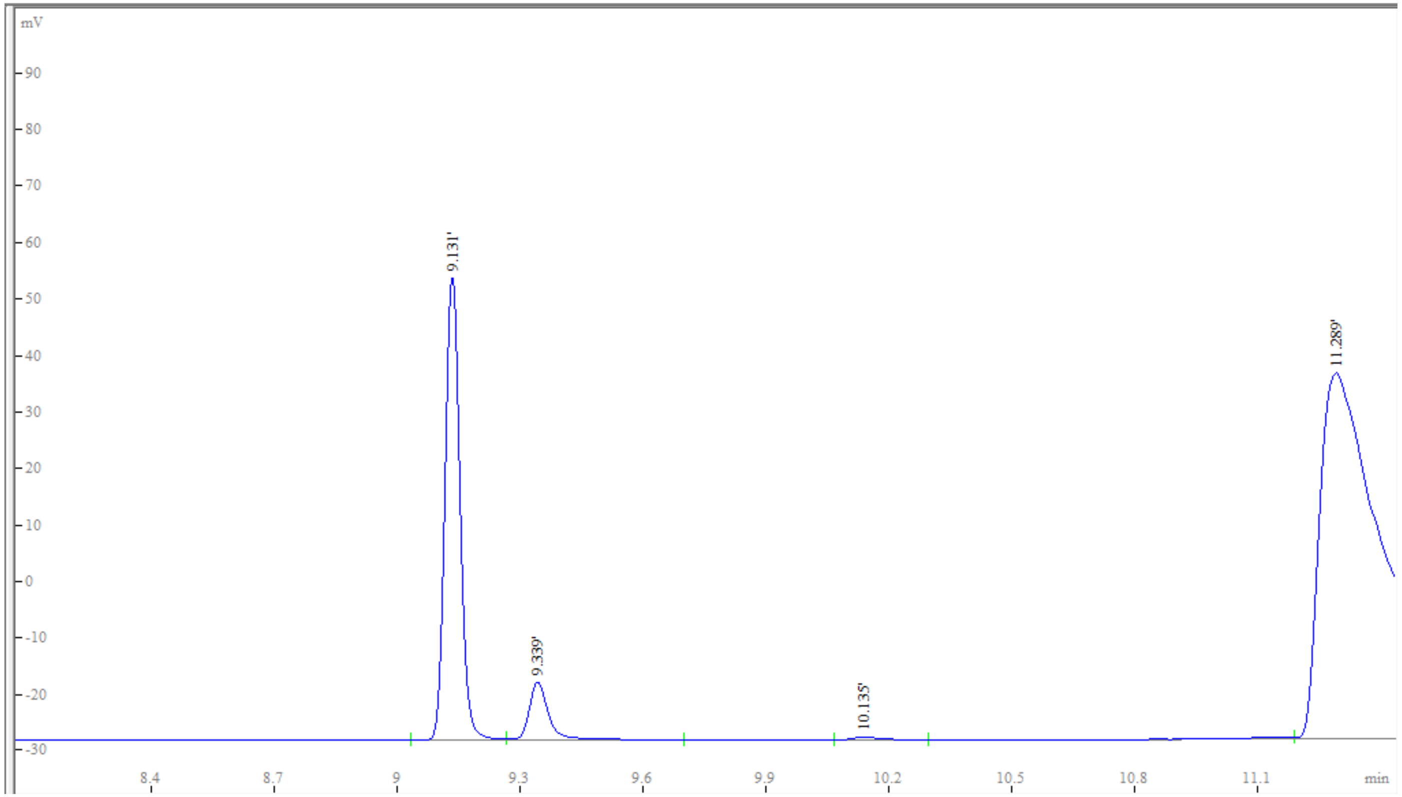

(c)

Figure S5. (a) A chromatogram of the reaction of entry 1 in table 2; (b) A chromatogram of the reaction of entry 2 in table 2; (c) A chromatogram of the reaction of entry 3 in table 2; (A) toluene; (B)2-iodobutane; (C)1-iodobutane; (D) o-xylene

2.2 Gas chromatography (additional experiment of table 1 with some modification)

The gas chromatography (G5, puri general instrument Co., Ltd., Beijing, China) equipped with FID detector and the capillary chromatographic column was **SE-30** (30m×0.32mm× 0.25μm). The GC operating condition was as follows: The injector was held at 200°C, The initial oven temperature of **100°C** was held for **6 min**, then which was increased to **200°C at 10°C/min**. This temperature was held for 4 min, too. The injection volume was 2 μL. The detector temperature was 250°C.

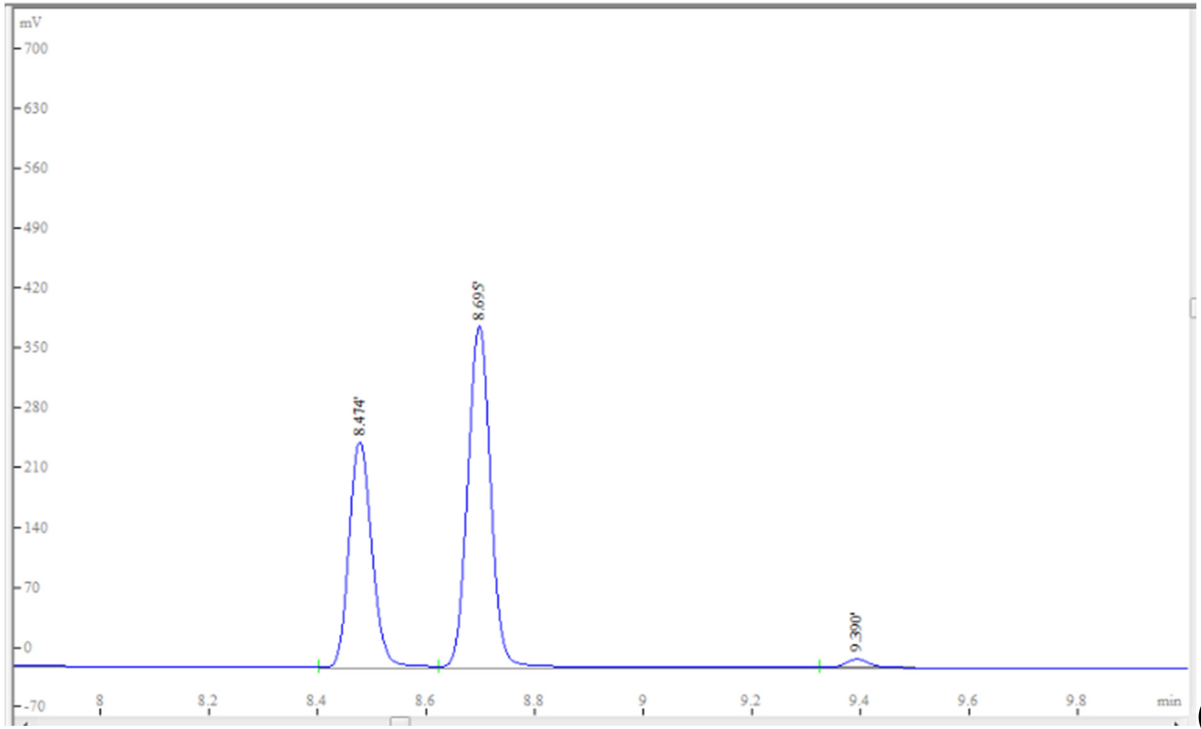

(a)

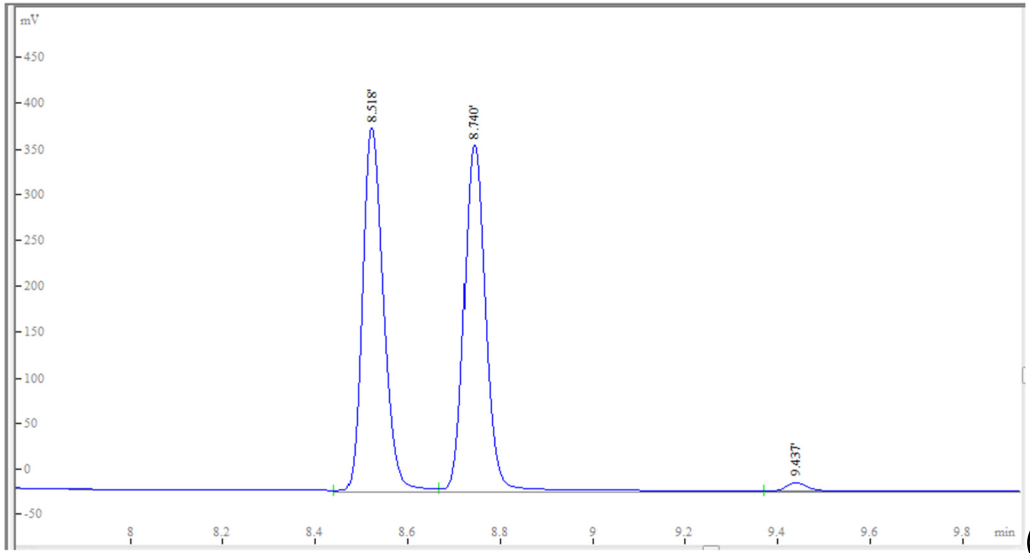
mV
450
400
350
300
250
200
150
100
50
0
-50
8.518'
8.740'
9.437'
8
8.2
8.4
8.6
8.8
9
9.2
9.4
9.6
9.8
min
(b)

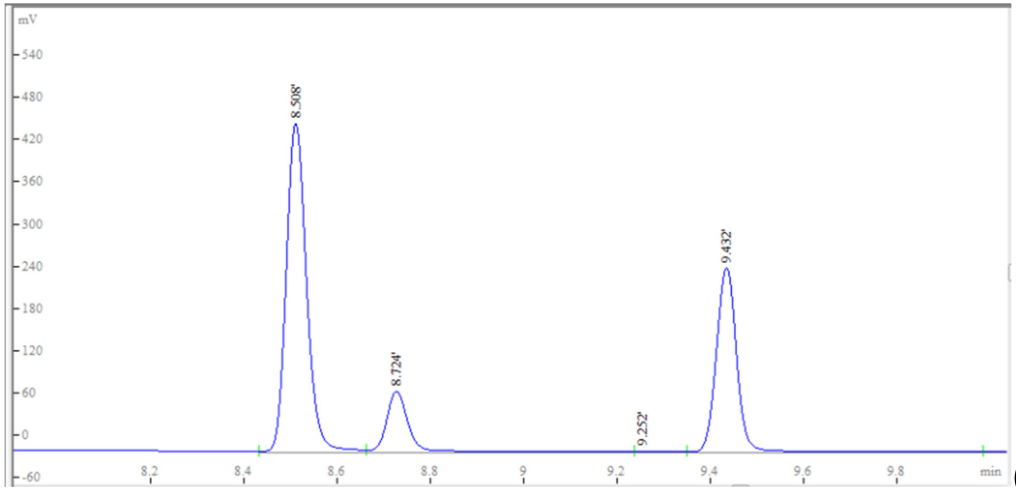
mV
540
480
420
360
300
240
180
120
60
0
-60
8.508'
8.724'
9.252'
9.432'
8.2
8.4
8.6
8.8
9
9.2
9.4
9.6
9.8
min
(c)

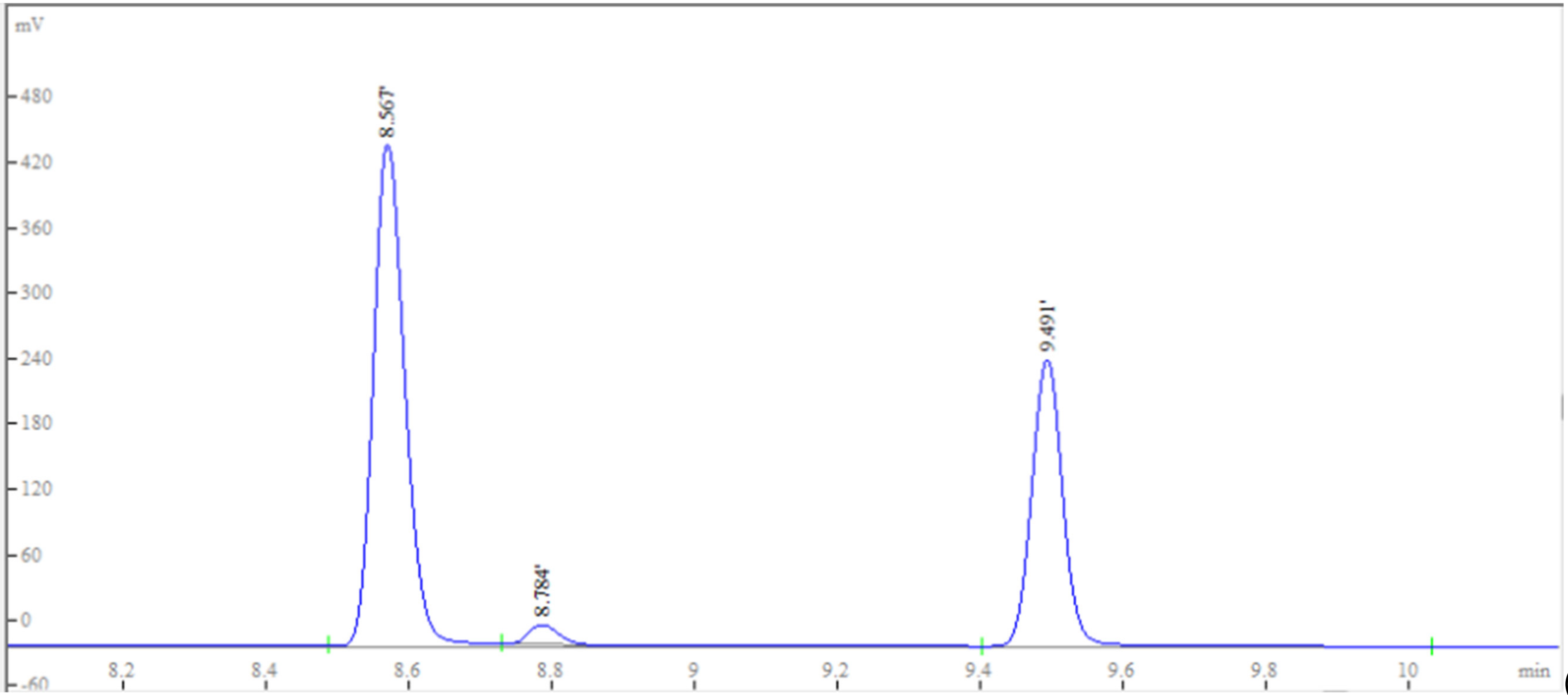
mV
480
420
360
300
240
180
120
60
0
-60
8.567
8.784
9.491
8.2
8.4
8.6
8.8
9
9.2
9.4
9.6
9.8
10
min
(d)

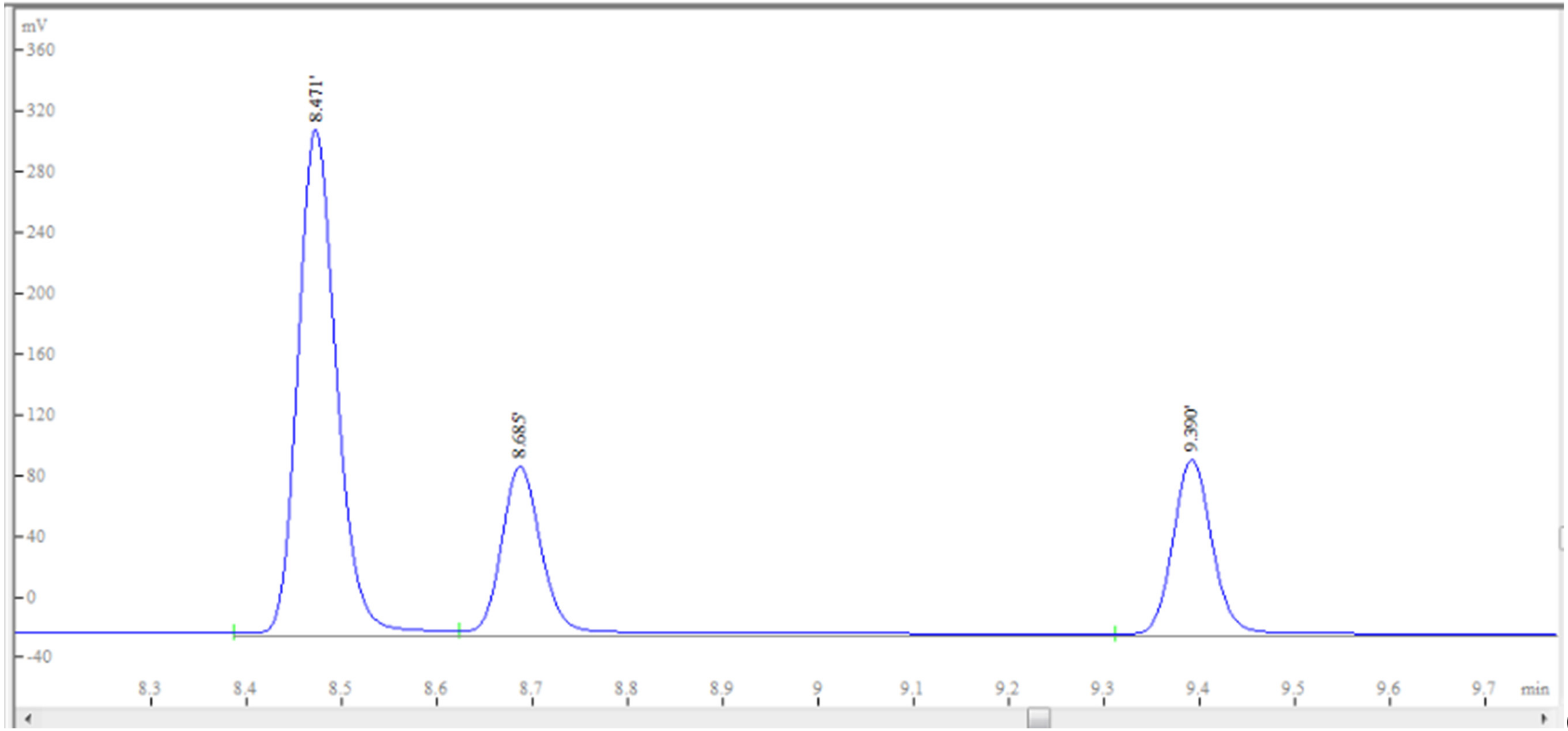

(e)

Figure S6. (a) A chromatogram of the reaction of entry 1 in table 1; (b) A chromatogram of the reaction of entry 2 in table 1; (c) A chromatogram of the reaction of entry 3 in table 1; (d) A chromatogram of the reaction of entry 4 in table 1; (e) A chromatogram of the reaction of entry 5 in table 1;
